# Single-Cell RNA Sequencing in Unraveling Acquired Resistance to EGFR-TKIs in Non-Small Cell Lung Cancer: New Perspectives

**DOI:** 10.3390/ijms26041483

**Published:** 2025-02-11

**Authors:** Lin Peng, Siyou Deng, Jinjie Li, Yujie Zhang, Li Zhang

**Affiliations:** Department of Oncology, Tongji Hospital, Tongji Medical College, Huazhong University of Science and Technology, Wuhan 430030, China; pennytj@hust.edu.cn (L.P.); m202376562@hust.edu.cn (S.D.); m202476654@hust.edu.cn (J.L.); tjzyj@tjh.tjmu.edu.cn (Y.Z.)

**Keywords:** single-cell RNA sequencing, non-small cell lung cancer, EGFR-TKI, acquired resistance, tumor microenvironment, heterogeneity

## Abstract

Epidermal growth factor receptor tyrosine kinase inhibitors (EGFR-TKIs) have demonstrated remarkable efficacy in treating non-small cell lung cancer (NSCLC), but acquired resistance greatly reduces efficacy and poses a significant challenge to patients. While numerous studies have investigated the mechanisms underlying EGFR-TKI resistance, its complexity and diversity make the existing understanding still incomplete. Traditional approaches frequently struggle to adequately reveal the process of drug resistance development through mean value analysis at the overall cellular level. In recent years, the rapid development of single-cell RNA sequencing technology has introduced a transformative method for analyzing gene expression changes within tumor cells at a single-cell resolution. It not only deepens our understanding of the tumor microenvironment and cellular heterogeneity associated with EGFR-TKI resistance but also identifies potential biomarkers of resistance. In this review, we highlight the critical role of single-cell RNA sequencing in lung cancer research, with a particular focus on its application to exploring the mechanisms of EGFR-TKI-acquired resistance in NSCLC. We emphasize its potential for elucidating the complexity of drug resistance mechanism and its promise in informing more precise and personalized treatment strategies. Ultimately, this approach aims to advance NSCLC treatment toward a new era of precision medicine.

## 1. Introduction

According to the latest estimates from the International Agency for Research on Cancer (IARC), nearly 20 million new cancer cases and 10 million cancer-related deaths were projected globally in 2022 [1]. These figures underscore that cancer has become one of the most pressing social and public health challenges of the 21st century. Among various cancers, lung cancer ranks as the most prevalent worldwide and is the leading cause of cancer-related mortality, drawing significant attention from scientists and medical professionals across the globe. Notably, non-small cell lung cancer (NSCLC), which accounts for approximately 85% of all lung cancer cases, presents a complex clinical profile and is often diagnosed in advanced stages, contributing to its poor prognosis [2]. In recent years, the rapid evolution of lung cancer treatment, especially the discovery of lung cancer driver genes, has significantly advanced the field of tumor therapy. A breakthrough came with the identification of mutations in the epidermal growth factor receptor (EGFR), heralding a new era of personalized cancer treatment. This discovery paved the way for *EGFR*-targeted therapies, with epidermal growth factor receptor tyrosine kinase inhibitors (EGFR-TKIs) becoming the standard first-line treatment for NSCLC [3,4,5]. This therapeutic approach has not only achieved notable progress in prolonging patient survival but also significantly improved the quality of life for individuals battling this disease.

The rapid emergence of acquired resistance significantly limits the effectiveness of EGFR-TKI therapy, while the inability to accurately identify biomarkers in NSCLC patients hampers the development of targeted and effective treatments. Acquired resistance typically arises through a variety of mechanisms, including secondary mutations and persistent EGFR activation, alterations in downstream signaling pathways, phenotypic transformations, modifications in the tumor microenvironment, and immune dysregulation [6,7,8]. Research has demonstrated that the *EGFR T790M* mutation is among the most prevalent mechanisms of acquired resistance to first- and second-generation EGFR-TKIs. This mutation restores the activated state of EGFR, leading to a significant reduction in the efficacy of the TKI. However, third-generation EGFR-TKIs, such as osimertinib, have shown sensitivity to this mutation [9,10,11]. Furthermore, changes within the tumor microenvironment have been identified as a key contributor to EGFR-TKI resistance, further constraining the therapeutic potential of relying solely on EGFR-TKIs [12]. The complexity and heterogeneity of resistance mechanisms within tumors significantly impact the therapeutic response to EGFR-TKI treatments in *EGFR*-mutant NSCLC [13]. Consequently, a deeper investigation into EGFR-TKI resistance mechanisms is critical for optimizing treatment strategies and delaying the onset of resistance.

In recent years, the rapid advancement of single-cell RNA sequencing technology has enabled researchers to explore tumor heterogeneity and the interactions within the tumor microenvironment at the resolution of individual cells. This cutting-edge technology not only captures the transcriptomic landscapes of tumor cells but also uncovers the interplay between different cell types in the tumor microenvironment, offering a fresh perspective on tumor drug resistance [14]. By shedding light on the genetic and phenotypic variability among tumor cells, researchers can better understand the diversity of tumor cell populations and their varied responses to treatment. This approach facilitates the acquisition of highly specific genetic information at the individual cell level, which is vital for advancing our understanding of tumor biology [15,16]. The implementation of this technology provides not only a transformative tool for uncovering the biological properties of cancer but also paves the way for novel avenues of research into the onset, progression, and mechanisms of drug resistance in NSCLC.

In summary, single-cell RNA sequencing technology offers distinctive advantages in tumor research. It enables a deeper understanding of the mechanisms underlying EGFR-TKI resistance and its interactions with the tumor microenvironment, while uncovering the complexity of tumor heterogeneity during drug resistance. This insight provides opportunities to identify novel resistance biomarkers and therapeutic targets, ultimately improving the prognosis and survival rates of NSCLC patients. This review highlights recent advancements in the application of single-cell sequencing in lung cancer research, with a particular focus on the mechanisms of acquired resistance to EGFR-TKIs in NSCLC. It also underscores the potential of multi-omics analysis based on single-cell sequencing data. This cutting-edge technology not only introduces innovative approaches to clinical treatment but also informs the trajectory of future research initiatives.

## 2. Applications and Challenges of Single-Cell Sequencing Technology in Lung Cancer Research

### 2.1. Advances in Single-Cell Sequencing Technology

Single-cell sequencing technology has advanced rapidly since its inception, becoming a vital tool for studying cellular heterogeneity and dynamic gene expression. It enables researchers to uncover differences among cell populations and their evolutionary relationships while extracting multidimensional information on the genome, transcriptome, and epigenome at the single-cell level [17]. This technology not only identifies the genomic sequences of individual cells and highlights differences in genetic material and proteins between cells but also deepens our understanding of the roles and functions of individual cells within their microenvironment [18]. Commonly employed single-cell sequencing methods include single-cell RNA sequencing (scRNA-seq), single-cell DNA sequencing, and single-cell epigenome sequencing. In cancer research, single-cell sequencing holds immense promise, particularly in revealing tumor cell heterogeneity, monitoring tumor progression, and identifying novel therapeutic targets. As the field evolves, innovative methodologies continue to emerge. For example, the development of spatial transcriptomics has allowed researchers to localize specific cell types and analyze their interactions within tissue sections, offering more intuitive insights into the spatial organization of cells [19]. Moreover, the integration of single-cell technologies with long-read sequencing has enabled the capture of more complex transcript information, uncovering cell-specific splicing patterns and genomic structural variations [20]. On the data analysis front, advancements in computational algorithms and bioinformatics tools have significantly enhanced the efficiency and precision of extracting biological insights from single-cell sequencing data [21,22]. These technological strides have not only improved the resolution and sensitivity of single-cell sequencing but have also expanded the toolkit available to life sciences and medical researchers. This progress has deepened our understanding of biology, opened new avenues for clinical applications, and ushered in exciting possibilities for the future of scientific discovery.

### 2.2. Single-Cell Sequencing in Lung Cancer Research

The advent of single-cell sequencing technology has transformed tumor research. This groundbreaking technology has been extensively applied to the study of various malignant tumors, including lung, colorectal, pancreatic, prostate, and breast cancers. It has enabled scientists to deepen their understanding of tumorigenesis, progression, and drug resistance, while uncovering critical clues for identifying novel biomarkers and developing innovative therapeutic strategies [23,24,25,26,27]. By exposing tumor heterogeneity, single-cell sequencing has the remarkable ability to pinpoint distinct tumor subpopulations and their associated driver mutant genes, thereby offering a more precise foundation for personalized treatments [14,28]. This section delves into the application of single-cell sequencing in lung cancer research, highlighting its distinctive value in identifying resistance-related cell subpopulations, dynamically unraveling resistance mechanisms, integrating multi-omics data, and advancing personalized treatment strategies. These novel insights pave the way for a more profound understanding of EGFR-TKI resistance mechanisms and open up innovative avenues for future treatment approaches in clinical practice.

#### 2.2.1. Identification of Resistance-Related Cell Subpopulations

Single-cell sequencing has showcased unparalleled advantages in identifying tumor cell subpopulations, offering novel methods for mapping tumor evolution. For instance, OncoNEM operates as a probabilistic tool to quantify variant copy numbers using single-cell sequencing data, uncovering genomic diversity and shedding light on the genetic heterogeneity of tumors. Moreover, OncoNEM can pinpoint molecular phenotypes and cellular subpopulations of tumor cells, enabling the construction of tumor evolution maps and the generation of tumor phylogenetic trees [29]. Sharma et al. [30] employed single-cell RNA sequencing to analyze lung squamous cell carcinoma tumors, identifying new clusters of tumor subclones and exposing differences in intra- and intertumor heterogeneity. Their findings highlight the role of tumor heterogeneity in influencing the evolutionary dynamics of tumors across various levels. Using scRNA-seq, researchers can identify drug resistance-associated cell subpopulations, such as drug-tolerant persister (DTP) cells. These cells persist following drug treatment and may serve as “seed cells” for resistance. Studies have revealed that DTP cells exhibit a distinct gene expression profile, characterized by the activation of genes linked to cell cycle arrest and metabolic reprogramming [31,32,33]. Guo et al. [34] conducted scRNA-seq studies on tumor cells, adjacent normal tissues, and peripheral blood T cells from patients with untreated lung adenocarcinoma and squamous carcinoma. Their work revealed significant differences in the composition and proportion of T cells across tissues, demonstrating heterogeneity in tumor-infiltrating lymphocytes and providing a potential new pathway for advancing tumor immunotherapy. Furthermore, scRNA-seq facilitates the identification of various subtypes of tumor-associated macrophages (TAMs) and their contributions to drug resistance. For instance, certain TAM subtypes may drive resistance by secreting immunosuppressive factors, such as IL-10, or by impeding T-cell infiltration [35,36]. Finally, a study using scRNA-seq to analyze EGFR-mutated early lung adenocarcinoma tissues and paraneoplastic normal lung cells unveiled the heterogeneity among malignant subpopulations. This heterogeneity manifested through inflammatory cytokine stimulation, such as IL-1B, and the upregulation of the *ELF3* gene, which activated the PI3K/Akt/NF-κB pathway, thereby enhancing the expression of tumor genes linked to proliferation and anti-apoptosis mechanisms [37].

Single-cell sequencing technology has proven to be a transformative tool in identifying tumor drug-resistant cell subpopulations, uncovering their heterogeneity and evolutionary dynamics within a tumor. Through the analysis of various tumor types, researchers have successfully pinpointed distinct cell subpopulations and highlighted their pivotal roles in tumor progression, drug resistance, and immune response. These findings offer a crucial foundation for advancing our understanding of tumor biology and developing personalized therapeutic strategies, while also deepening insights into the mechanisms of tumorigenesis and evolution.

#### 2.2.2. Dynamic Analysis of Drug Resistance Mechanisms

The application of single-cell sequencing in exploring lung cancer drug resistance mechanisms has been demonstrated using several real-world samples. By utilizing scRNA-seq, researchers can monitor the dynamic changes in tumor cells during EGFR-TKI treatment. For instance, studies have demonstrated that following EGFR-TKI therapy, tumor cells transition into a drug-tolerant persistent state, characterized by distinct gene expression profiles, including the upregulation of the anti-apoptotic protein BCL-XL [38]. These dynamic adaptations shed light on the evolution of resistance mechanisms. Furthermore, scRNA-seq offers insights into the shifting quantity and functional status of immune cells within the tumor microenvironment over time, aiding the identification of novel biomarkers [39]. As an example, in EGFR-TKI resistant tumors, tumor-associated macrophages (TAMs) undergo significant polarization, transitioning from the pro-inflammatory M1 phenotype to the immunosuppressive M2 phenotype. This cellular remodeling plays a pivotal role in advancing our understanding of resistance mechanisms [40]. Ma et al. [41] demonstrated that heterogeneous expression of genes involved in the IFN-γ signaling pathway was associated with the development of an acquired resistance phenotype in individual cancer cells. Their findings suggested that the downregulation of this signaling pathway may contribute to the emergence of drug resistance, further advancing our understanding of resistance mechanisms at the molecular level.

Single-cell sequencing technology enables the dynamic analysis of expression differences between responders and non-responders to targeted lung cancer therapies. By examining these differentially expressed genes or specific cell subpopulations, notable distinctions in expression patterns between the two groups can be identified. Such differences hold promise as potential biomarkers, offering valuable insights into the mechanisms of drug resistance and paving the way for more personalized treatment approaches [31,42,43,44,45,46].

#### 2.2.3. Integration of Multi-Omics Data

While single-cell RNA sequencing offers transcriptomic insights at the single-cell level, its primary limitation lies in the absence of spatial information. Integrating spatial transcriptomics (ST) addresses this shortcoming by providing data on the spatial distribution of cells, enabling a more comprehensive understanding of resistance mechanisms [47]. For instance, one study combined scRNA-seq and ST data to uncover both intra- and inter-tumor heterogeneity in mouse *EGFR*-mutant lung cancer lesions [38]. To further bridge the gap in understanding patient-specific heterogeneity, it is essential to extend integration methods to include patient-level data. This approach allows for the identification of shared and unique resistance patterns among patients [48]. Moreover, since scRNA-seq captures only RNA expression levels—and not all RNAs are translated into proteins—advancements like CITE-seq have emerged. These technologies enable the simultaneous detection of the transcriptome and proteome within single cells, offering a more precise depiction of cellular states [49].

#### 2.2.4. Exploration of Personalized Treatment Strategies

Single-cell sequencing technology has demonstrated remarkable potential in predicting treatment responses. Unlike traditional tumor genome analysis, which typically offers gene expression data for the tumor as a whole, single-cell sequencing overcomes the limitations of revealing intra-tumor heterogeneity and the diverse responses to treatment [14]. By providing detailed gene expression profiles for individual cells, researchers can identify specific cell populations sensitive to particular therapies, while pinpointing subpopulations that may contribute to treatment resistance [28]. Additionally, this technology has been instrumental in identifying biomarkers linked to immunotherapy, aiding in the prediction of patient responsiveness to immune checkpoint inhibitors [50,51]. One study employed single-cell RNA sequencing to analyze the immune responses of NSCLC patients treated with a combination of pembrolizumab and chemotherapy in the neoadjuvant setting. The findings highlighted several pivotal immunological processes tied to therapeutic efficacy. Specifically, the study revealed that an increase in tertiary lymphoid structures within tumor lesions enhanced synergy between B cells and CD4+ T cells and that the conversion of B cells into an anti-tumor IgG class significantly amplified the anti-tumor immune response [52]. Single-cell RNA sequencing offers a powerful tool for identifying gene expression patterns and cell subpopulations associated with drug resistance. By examining the gene expression profiles of resistant cells, it enables the prediction of patient responses to specific therapies and aids in designing effective combination treatment strategies. For instance, research has demonstrated that BCL-XL plays a pivotal role in resisting EGFR-TKIs, and its inhibition has been shown to effectively overcome resistance to osimertinib [38].

By unveiling the gene expression profiles and heterogeneity of tumor cells, single-cell sequencing enables the identification of cell populations sensitive to treatment, as well as subpopulations responsible for treatment resistance. This breakthrough forms a critical foundation for personalized therapy and the optimization of immunotherapy, while aiding in the prediction of patient responses to various treatments. Looking ahead, single-cell sequencing is poised to emerge as an essential tool in precision medicine, enhancing treatment effectiveness and elevating the accuracy of clinical decision-making.

### 2.3. Advantages and Limitations of Single-Cell Sequencing in EGFR-TKI Resistance Research

The strength of single-cell sequencing technology in EGFR-TKI resistance research lies in its ability to deliver precise information at the cellular level, uncovering intercellular heterogeneity and dynamic changes that traditional bulk sequencing methods cannot achieve [14]. For instance, single-cell sequencing has revealed that lung adenocarcinoma can undergo transdifferentiation into squamous cell carcinoma under the selective pressure of EGFR-TKI treatment. This process is strongly linked to drug resistance, offering further evidence—at the single-cell level—that targeted therapy can drive squamous cell carcinoma transdifferentiation in lung cancer [53]. In contrast, conventional high-throughput sequencing approaches are limited to providing average gene expression profiles across cell populations, making it impossible to thoroughly analyze the variations in gene expression among individual cells. This inherently overlooks the genomic and transcriptomic diversity commonly observed in tumor cells. Particularly in EGFR-TKI resistance research, single-cell sequencing not only enables the effective classification of cell populations and the detection of subpopulations related to resistance but also provides an accurate depiction of tumor evolution. This capability offers crucial theoretical support for early cancer screening and the development of personalized treatment strategies [54,55]. With ongoing technological advancements, single-cell sequencing has found extensive applications in diverse research disciplines such as developmental biology, immunology, and oncology. These applications have significantly advanced the fields of single-cell genomics, epigenomics, and proteomics [56,57].

However, the application of single-cell sequencing technology in EGFR-TKI resistance research is not without its challenges. Currently, the technique is susceptible to amplification bias during the nucleotide amplification step, which often results in non-specific outcomes. Additionally, it is predominantly suited for use with suspended cellular fluids, making it difficult to accurately analyze the spatial context of cells or tissues [58]. Beyond these technical challenges, the sparse availability of resistant samples obtained from patients’ bodies significantly hampers the broader clinical adoption of scRNA-seq. The dynamic nature of resistance necessitates sampling at multiple time points; however, the repeated procedures required to collect these samples may pose risks and potential harm to patients. Nonetheless, leveraging single-cell sequencing in animal model studies offers a viable solution to address the issue of limited cell quantities, while subsequent validation in population cohorts ensures robustness. As such, the potential of scRNA-seq in advancing resistance research remains highly promising.

## 3. Mechanisms of Acquired Resistance to EGFR-TKIs in NSCLC

**Figure 1:**
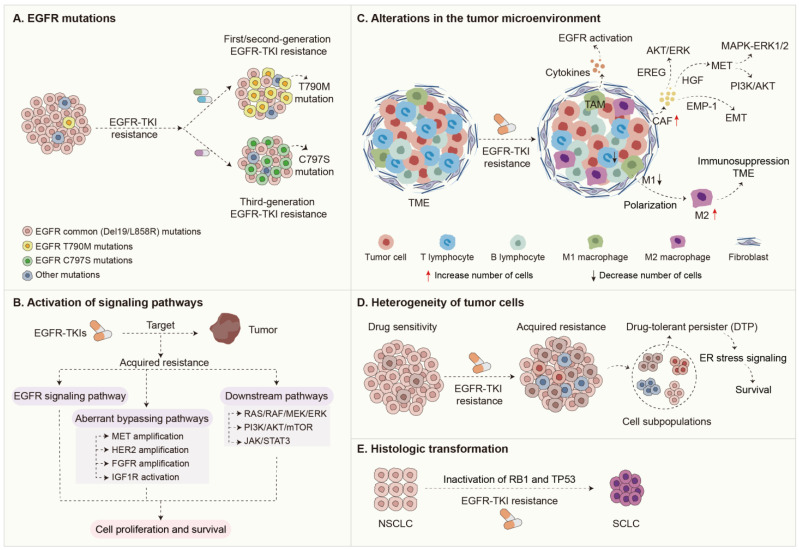
Mechanisms of acquired resistance to EGFR-TKIs in NSCLC.

The mechanisms underlying EGFR-TKI resistance are intricate and multifaceted, encompassing factors such as *EGFR* mutations, activation of signaling pathways, alterations in the tumor microenvironment, tumor heterogeneity, and histological transformation (Figure 1). These mechanisms not only illustrate the adaptive responses of tumor cells under the selective pressure of drug treatment but also highlight the crucial role of the tumor microenvironment in regulating therapeutic outcomes. The development of EGFR-TKI resistance is a complex, dynamic process shaped by various interrelated factors, ranging from genetic mutations to cellular behavior and immune responses.

### 3.1. EGFR Mutations and Signaling Pathway Activation

*EGFR* mutations play a critical role in determining treatment response in NSCLC patients. The most common types of *EGFR* mutations are the exon 19 deletion and the exon 21 L858R point mutation, both of which are typically associated with heightened sensitivity to EGFR-TKI therapy [59,60]. However, as treatment progresses, patients frequently develop acquired resistance. A primary mechanism underpinning this resistance is the emergence of secondary mutations, with the *EGFR T790M* mutation being the most prevalent. This mutation diminishes the drug’s inhibitory efficacy by enhancing the mutant receptor’s affinity for ATP [61,62,63]. Research indicates that approximately 45% to 65% of patients receiving first-generation (e.g., erlotinib, gefitinib) or second-generation (e.g., afatinib, dacomitinib) EGFR-TKIs may develop *T790M* mutations, leading to treatment failure [9,64,65]. The advent of third-generation EGFR-TKIs, such as osimertinib, has offered renewed therapeutic hope for patients with *EGFR* mutation-positive NSCLC. Nonetheless, emerging studies have identified the *EGFR C797S* mutation as a key mechanism of resistance to these third-generation therapies. This mutation fosters tumor progression by activating downstream signaling pathways [66,67].

Acquired resistance to EGFR-TKIs is primarily driven by the persistent activation of the EGFR signaling pathway [59]. Research indicates that tumor cells bypass EGFR-TKI suppression by reactivating EGFR and its downstream signaling cascades through various mechanisms. For instance, TFF3 promotes EGFR activation by positively regulating YAP, thereby contributing to drug resistance in *EGFR*-mutated lung adenocarcinomas [6]. This sustained signaling pathway activation not only compromises drug efficacy but also enhances tumor cell proliferation and metastasis, further exacerbating disease progression. Moreover, alterations in the tumor microenvironment can also sustain EGFR pathway activation. Tumor-associated macrophages, through the secretion of pro-inflammatory factors, may amplify EGFR activation within the tumor microenvironment, rendering tumor cells resistant to EGFR-TKI therapy [12].

In addition, aberrant activation of bypass pathways is a significant contributor to acquired resistance to EGFR-TKIs [68]. Research has revealed that *MET* amplification and *HER2* activation serve as key mechanisms driving resistance to second-generation EGFR-TKIs. These alterations allow tumor cells to bypass the EGFR signaling pathway, thereby enhancing their resistance to TKIs [69]. Currently, targeting MET in combination with EGFR-TKIs has emerged as a crucial therapeutic approach for treating EGFR-TKI-resistant NSCLC caused by *MET* amplification [70,71]. Furthermore, *HER2* amplification has been shown to foster osimertinib resistance by influencing the PI3K/AKT and MAPK signaling pathways [72,73]. Beyond these mechanisms, aberrant activation of fibroblast growth factor receptor (FGFR) signaling and the insulin-like growth factor 1 receptor (IGF1R) has also been closely linked to EGFR-TKI resistance [74,75,76,77].

Thus, the mechanisms underlying EGFR-TKI resistance are not limited to secondary mutations resulting from *EGFR* alterations; they also encompass the persistent activation of the EGFR signaling pathway and the aberrant activation of bypass pathways. These mechanisms are intricately linked, collectively driving the progression of drug resistance. Gaining a deeper understanding of these pathways offers valuable insights for clinical treatment. Future research should prioritize strategies to enhance both the efficacy and the durability of therapies by effectively targeting these resistance mechanisms, thereby improving patient outcomes and prognosis.

### 3.2. Alterations in the Tumor Microenvironment

The tumor microenvironment (TME) constitutes a highly intricate system shaped by the interplay between tumor cells, surrounding cellular components, and the stromal framework. The development of acquired drug resistance is influenced not only by gene mutations and the aberrant activation of signaling pathways within cancer cells but also by the complex interactions among diverse cellular and molecular factors within the TME.

Tumor-associated macrophages (TAMs) play a pivotal role in the development of EGFR-TKI resistance. By secreting a range of cytokines and chemokines, TAMs activate bypass pathways that promote tumor cell survival and proliferation, ultimately contributing to EGFR-TKI resistance [78]. For instance, tumor epithelial cells, fibroblasts, and macrophages in the tumor microenvironment produce EREG through both autocrine and paracrine mechanisms. EREG, in turn, stimulates tumor-promoting signals, leading to EGFR-TKI resistance in NSCLC cells [79]. Additionally, elevated expression of CCL2 in gefitinib-resistant cell lines facilitates the recruitment of M2-type macrophages and activates the AKT/mTOR signaling pathway, thereby diminishing gefitinib’s anti-tumor efficacy [80]. In early-stage tumors, TAMs are primarily of the M1 phenotype. However, within the tumor microenvironment of EGFR-TKI-resistant cancers, TAMs tend to polarize toward the immunosuppressive M2 phenotype. M2-type TAMs play a pivotal role in shaping the tumor immune microenvironment by upregulating immunosuppressive surface proteins, such as PD-L1, and secreting anti-inflammatory factors, including IL-10, TGF-β, and IL-4. These M2-type TAMs inhibit the function of effector T cells and promote the development and activation of regulatory T cells (Tregs), thereby facilitating tumor immune evasion and resistance [81]. Moreover, another study revealed that M2-type reprogramming of TAMs, coupled with the upregulation of CD47 in gefitinib-resistant lung cancer cells and tumor xenograft models, enables cancer cells to evade macrophage-mediated phagocytosis [40]. This further intensifies drug resistance. In resistant tumor cells, CD47 expression is markedly upregulated. CD47 interacts with SIRPα on the surface of macrophages, effectively suppressing macrophage-mediated phagocytosis of tumor cells. Research has further revealed that the excessive activation of STAT3 drives CD47 expression by binding to the promoter and intronic regions of the CD47 gene. Consequently, targeting STAT3 inhibition or employing anti-CD47 monoclonal antibodies can enhance TAM-mediated phagocytosis of tumor cells, thereby mitigating EGFR-TKI resistance.

Moreover, the immune response within the tumor microenvironment significantly influences the therapeutic efficacy of EGFR-TKIs. Research indicates that adaptive immune activation plays a crucial role in enhancing the effectiveness of EGFR-TKIs [82]. However, macrophage-induced T-cell suppression within the tumor microenvironment can diminish the therapeutic impact of osimertinib, while the establishment of an immunosuppressive TME facilitates the development of resistance to EGFR-TKIs [83]. EGFR-TKI therapy not only activates the immune response in *EGFR*-mutant NSCLC but also leads to increased immune cell infiltration within the tumor microenvironment following the onset of resistance. This infiltration triggers TME remodeling, further intensifying therapeutic resistance [84]. Research indicates that in the tumor microenvironment resistant to EGFR-TKIs, the expression of immune checkpoint molecules, such as PD-L1, may undergo significant alterations. These changes can affect the infiltration and functionality of immune cells, consequently facilitating immune evasion [85]. As a result, combining EGFR-TKIs with immune checkpoint inhibitors, such as PD-1/PD-L1 inhibitors, holds potential for remodeling the immune microenvironment and strengthening the anti-tumor immune response.

The role of fibroblasts within the tumor microenvironment is critical. Cancer-associated fibroblasts (CAFs) represent one of the predominant cell types within the tumor microenvironment, playing a pivotal role in regulating tumor cell behavior by releasing growth factors, chemokines, and extracellular matrix (ECM) components. In tumors resistant to EGFR-TKIs, CAFs contribute to resistance mechanisms by secreting specific cytokines, such as TGF-β, and various growth factors [86]. Wang et al. [87] discovered that hepatocyte growth factor (HGF) secreted by stromal fibroblasts restores downstream MAPK-ERK1/2 phosphorylation by activating the MET receptor, thereby contributing to intrinsic resistance to EGFR-TKIs. Moreover, HGF was found to activate the PI3K/Akt signaling pathway in *EGFR*-mutant lung adenocarcinoma, facilitating tumor cell proliferation. Additionally, CAFs exhibit primary resistance to EGFR-TKIs in *EGFR*-mutant lung adenocarcinoma. Research has demonstrated that silencing the expression of podoplanin in CAFs can effectively eliminate the resistance of tumor cells to EGFR-TKIs [88]. Similarly, Mink et al. [86] observed that CAFs undergoing epithelial–mesenchymal transition (EMT) in EGFR-TKI-resistant tumors expressed elevated levels of the gefitinib-resistant biomarker epithelial membrane protein-1 (EMP-1). This finding represented the first demonstration that the tumor stroma undergoes modifications associated with the acquisition of EGFR-TKI resistance, which further drives the development of drug resistance. These EMT-derived CAFs demonstrate an enhanced capacity to drive resistance and, in collaboration with host fibroblasts, effectively hinder the inhibition of the EGFR pathway by EGFR-TKIs. The tumor microenvironment also fosters EMT by creating a conducive setting for cancer cell survival and proliferation, ultimately mediating resistance to EGFR-TKIs in NSCLC [89,90,91]. Consequently, targeting CAFs or their secreted factors represents a promising therapeutic strategy to overcome EGFR-TKI resistance.

In summary, tumor-associated macrophages, immune responses, and fibroblasts within the tumor microenvironment play pivotal roles in the development of EGFR-TKI resistance. A deeper exploration of the intricate mechanisms governing the tumor microenvironment may offer innovative therapeutic strategies to overcome EGFR-TKI resistance as well as identify potential targets for future clinical treatments.

### 3.3. Heterogeneity of Tumor Cells

Tumor cell heterogeneity encompasses the genetic, phenotypic, and behavioral variations among cells within the same tumor. This heterogeneity plays a pivotal role in driving the development of resistance to EGFR-TKIs, resulting in varying drug responses across different cell populations [92,93].

After the development of resistance to EGFR-TKI treatment, tumor cells undergo significant alterations in their gene expression profiles. Research indicates that genes associated with cell proliferation, apoptosis, and metabolism exhibit markedly changed expression levels in drug-resistant cells and that such changes may contribute to the cells’ resistance to therapy [94]. In NSCLC, genome sequencing and transcriptome analyses of tumor tissues have uncovered notable molecular distinctions among various tumor subtypes. The molecular profiling of tumor cells demonstrates extensive tumor heterogeneity, which plays a critical role in the emergence of EGFR-TKI resistance [95]. Understanding the mechanisms underlying tumor heterogeneity is essential for overcoming resistance to EGFR-TKI therapy. For instance, growing evidence indicates that *APOBEC* mutations are prevalent in acquired EGFR-TKI-resistant NSCLC, underscoring the role of APOBEC enzymes in the evolution of *EGFR*-mutant lung cancer and their potential as therapeutic targets for addressing tumor heterogeneity [96,97]. Research has demonstrated that inhibiting the EGFR oncogene activates the NF-κB signaling pathway in lung cancer, subsequently increasing the expression of the apolipoprotein B mRNA-editing catalytic polypeptide-like (APOBEC) enzyme APOBEC3B (A3B) [98]. Analysis of single-cell RNA sequencing data from NSCLC clinical samples reveals that in tumors undergoing EGFR-TKI treatment, the expression of *A3B* mRNA, along with NF-κB components RELA and RELB, rises significantly, particularly during the disease progression phase. These findings propose that combining EGFR-TKI therapy with NF-κB inhibitors may represent a promising strategy to delay or prevent resistance development. Moreover, the phenotypic characteristics and behaviors of tumor cells are known to change dynamically. Epithelial–mesenchymal transition, for instance, is a pivotal process by which drug-resistant tumor cells may transform into more aggressive cell types that evade drug inhibition. The EMT program is characterized by heightened expression of inflammatory signals, IFN-γ, various co-inhibitory and co-stimulatory molecules, as well as increased infiltration of CD8+ and CD4+ T cells, regulatory T cells (Tregs), and macrophages. Research indicates that in NSCLC with high PD-L1 expression, EMT is linked to elevated infiltration of immunosuppressive cells, such as M2 macrophages and Tregs, while the presence of cytotoxic T lymphocytes (CTLs) declines [99]. Furthermore, cytokines released by tumor cells during EMT, such as TGF-β, can drive the polarization of TAMs toward an immunosuppressive M2 phenotype, facilitating immune evasion [100]. A study by Soucheray et al. [101] highlights the heterogeneity observed within NSCLC cell lines harboring EGFR mutations, which leads to distinct mechanisms of drug resistance. Their findings reveal that EGFR inhibition triggers TGF-β secretion, activating the SMAD pathway and promoting EMT, thereby further facilitating drug resistance. These studies underscore the varied responses and resistance mechanisms among different tumor cell subpopulations to EGFR-TKI therapy.

Cancer cell subpopulations demonstrate notable heterogeneity in their responses to drug therapy, particularly in the case of drug-tolerant persister (DTP) subpopulations. These DTP cells evade the cytotoxic effects of drugs by dynamically regulating their phenotypic heterogeneity, thereby shielding certain cells from drug inhibition [102]. DTP cells display considerable heterogeneity in gene expression and epigenetic modifications, enabling them to adapt to varying drug pressures and subsequently reactivate and proliferate following treatment [103]. While the majority of cell populations are effectively eradicated by drugs, DTP cells preserve their resistance through the coordinated action of multiple mechanisms. These include molecular modification regulation, alterations in signaling pathways, changes in the tumor microenvironment, metabolic reprogramming, and maintenance of redox balance. Together, these interconnected mechanisms allow DTP cells to withstand drug treatments, ultimately contributing to the emergence of resistance. Terai et al. [104] identified that DTP cells enhance survival through ER stress signaling, which, in turn, mediates their resistance to EGFR-TKIs. Furthermore, homologous frameshift DNA methylation has been implicated as a novel tumor cell state involved in intrinsic resistance to EGFR-TKI therapy. This discovery further highlights the pivotal role of tumor heterogeneity in the development of acquired resistance to EGFR-TKIs [105].

Most contemporary studies on the mechanisms of EGFR-TKI resistance have primarily focused on the outcomes of pre-existing clonal selection. Consequently, it is vital to examine the dynamic changes in tumor subclones and their influence on tumor biology and cancer progression [106]. Researchers have investigated the heterogeneity of *EGFR* mutation-positive NSCLC tumor cells through the lens of clonal evolution, proposing that EGFR-TKI resistance is intricately linked to a high degree of intracellular heterogeneity. This insight has provided a molecular foundation for understanding the resistance mechanisms [107]. Evidence suggests that, under the selective pressure of EGFR-TKI treatment, distinct tumor cell subpopulations may undergo selective expansion, allowing subclones harboring resistance-associated mutations to persist and emerge as dominant tumor clones. Furthermore, it has been observed that certain cell subpopulations with strong DDX3X expression exhibit stem cell-like properties and features associated with EMT. Notably, these subpopulations lack EGFR signaling and display significant resistance to EGFR-TKIs. These findings suggest that targeting DDX3X could represent a promising strategy to overcome primary resistance to EGFR-TKIs driven by intra-tumor heterogeneity [108].

In conclusion, tumor cell heterogeneity plays a pivotal role in driving the development of EGFR-TKI resistance through various mechanisms. These include alterations in gene expression, dynamic shifts in phenotype and behavior, the presence of drug-resistant persistent cell populations, and clonal evolution. Real-time monitoring of the molecular characteristics of tumors, coupled with dynamic tracking of drug-resistant gene expression and mutations, enables the timely detection of early signs of resistance during EGFR-TKI therapy.

### 3.4. Histological Transformation

Histological transformation into small cell lung cancer (SCLC) represents a significant mechanism of acquired resistance to EGFR-TKI therapy in patients with NSCLC [109,110,111]. Lee et al. [112] demonstrated that EGFR-TKI-resistant lung adenocarcinoma and SCLC tissues share a common clonal origin and undergo branching evolution, as revealed through analysis of both tissue types. Their findings indicate that the inactivation of TP53 and RB1 at the initial diagnosis of NSCLC is strongly associated with histological transformation. This suggests that genetic abnormalities in these genes might drive the progression of NSCLC into SCLC, ultimately contributing to EGFR-TKI resistance. While current research on the mechanisms underlying histological transformation remains limited, further investigation in this area is urgently needed. Early identification of tumor histological transformation is essential to facilitate timely adjustments to treatment strategies.

## 4. Application of scRNA-Seq to Reveal the Mechanisms of Acquired Resistance to EGFR-TKIs in NSCLC

**Figure 2:**
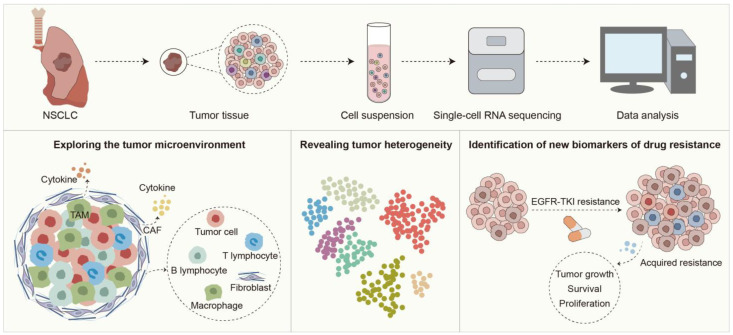
Application of scRNA-seq to reveal the mechanisms of EGFR-TKI resistance in NSCLC.

The mechanisms of EGFR-TKI resistance are complex and diverse, and traditional molecular biology methods are difficult to comprehensively reveal their underlying causes. Through high-resolution single-cell analysis, scRNA-seq is able to comprehensively explore the dynamic changes of tumor cells and their microenvironment, reveal drug resistance-related cell subpopulations and their molecular features, and identify drug resistance biomarkers, thus helping us to gain a deeper understanding of the multidimensional mechanisms of EGFR-TKI resistance (Figure 2).

### 4.1. Exploring the Tumor Microenvironment

In recent years, tumors have increasingly been conceptualized as a tumor ecosystem, wherein interactions between tumor cells, neighboring cells, and host cells enable the tumor to adapt and evolve, effectively evading immune surveillance and therapeutic interventions [113]. The emergence of single-cell RNA sequencing technology has equipped researchers with an unprecedented ability to conduct genomic and transcriptomic analyses at the single-cell level. This advancement has facilitated a more profound understanding of the dynamic changes within the tumor microenvironment and the evolutionary processes driving tumor cell growth [114,115].

Maynard et al. [48] conducted scRNA-seq assays on biopsy samples from metastatic lung cancer patients at various stages of therapy—prior to targeted treatment, after treatment initiation, and upon the development of drug resistance. Their study highlighted the abundance and diversity of mutations and transcripts within individual tumor samples. It also captured the dynamic changes in transcriptional profiles and the TME composition during the course of treatment. Notably, the research uncovered significant shifts in the composition and function of immune cells within the TME following the onset of drug resistance. Specifically, tumor-associated macrophages and cancer-associated fibroblasts exhibited dynamic changes, potentially contributing to the formation of an immunosuppressive TME and enabling tumor immune escape [12]. For instance, single-cell RNA sequencing was leveraged to examine changes in specific immune cell subpopulations within the TME of *EGFR*-mutant NSCLC patients who developed resistance after treatment with EGFR-TKIs. This analysis identified SLC40A1-positive macrophages as key players in the progression of resistance, suggesting they remodel the TME to foster immunosuppression [116]. Similarly, Han et al. [117] investigated TME reprogramming mechanisms using single-cell transcriptome sequencing of tumor tissues from osimertinib-resistant transplantation tumors. Their findings demonstrated that combination therapy with anlotinib significantly increased the infiltration of CD4+ T cells, CD25+CD4+ T cells, and PD-1+CD8+ T cells, enhancing the anti-tumor immune response. The study further analyzed cell subpopulation composition and function in the TME, unveiling an increase in immunosuppressive TAM.mo subtypes as a driving factor in creating an inhibitory immune microenvironment. This immunosuppressive state not only compromised the functionality of immune cells but also diminished the efficacy of EGFR-TKI therapy, providing an insight into a potential mechanism underlying osimertinib resistance.

In addition to the role of TAMs, other cells and components within the TME significantly contribute to the development of EGFR-TKI resistance. Wang et al. [118], through single-cell sequencing data analysis of advanced NSCLC patients before and after targeted therapy, discovered that CAFs became enriched during disease progression. These CAFs facilitated tumor EMT and enhanced tumor invasion by secreting an abundance of extracellular matrix proteins, ultimately leading to EGFR-TKI resistance. Moreover, studies integrating scRNA-seq data identified that the infiltration of CXCR1+ neutrophils—capable of promoting tumor cell resistance to third-generation EGFR-TKIs via activation of the TNF-α/NF-κB signaling pathway—was closely associated with EGFR-TKI resistance [119]. Conversely, Wang et al. [85] demonstrated that the inhibitory immune checkpoint PD-L2 underwent dynamic changes during EGFR-TKI treatment resistance. Elevated PD-L2 expression significantly impaired CD8+ T cell-mediated apoptosis of tumor cells, thereby driving alterations in the tumor immune microenvironment.

These studies highlight the capability of single-cell RNA sequencing technology to uncover the intricate and dynamic changes within the TME during EGFR-TKI resistance. In particular, it sheds light on the alterations in the tumor immune microenvironment and their connection to immune escape mechanisms. Single-cell RNA sequencing offers an unparalleled ability to uncover the composition, relative abundance, and interactions of various cell types within the tumor microenvironment at single-cell resolution. This technology enables researchers to map the dynamic shifts in immune cell populations throughout tumor progression, from their early accumulation in initial stages to their gradual buildup in advanced tumors. Furthermore, single-cell RNA sequencing can trace the developmental trajectories of specific cells, revealing transitions in cell states and identifying pivotal points within the EMT process. This high-resolution approach provides valuable insights into the dynamic patterns of immune cell infiltration in NSCLC, offering a deeper understanding of the tumor’s evolution and immune response. This advancement offers a fresh perspective for understanding the molecular basis of resistance to targeted therapies and holds the potential to inspire novel diagnostic and therapeutic strategies to address such resistance.

### 4.2. Revealing Tumor Heterogeneity

Tumor heterogeneity plays a critical role in the development of EGFR-TKI resistance in EGFR-mutated NSCLC. A comprehensive understanding of the mechanisms driving tumor heterogeneity in TKI resistance is pivotal for advancing more effective targeted therapies. For instance, Lambrechts et al. [120] discovered significant inter-individual differences in the stromal cells of NSCLC through scRNA-seq, highlighting a level of heterogeneity that may influence therapeutic efficacy and contribute to the emergence of drug resistance mechanisms. Research has demonstrated that tumor cell heterogeneity manifests not only between patients but also within distinct tumor cell populations of a single patient, thereby complicating the study of drug resistance mechanisms [121]. Maynard et al. [48] utilized scRNA-seq to analyze biopsy specimens from advanced NSCLC patients at multiple time points—both before and after the administration of targeted therapies—unveiling extensive intra-tumoral heterogeneity. This discovery underscores the potential of tailoring therapies to specific cellular subpopulations associated with resistance mechanisms to enhance patient survival outcomes. Furthermore, a prospective study on osimertinib-resistant NSCLC patients with EGFR mutations identified various molecularly driven resistance mechanisms through scRNA-seq. It also highlighted the clonal evolution heterogeneity of tumor cells as a key factor driving disease progression [122]. Researchers have utilized scRNA-seq not only to identify tumor heterogeneity in resistant cell line models and clinical specimens with acquired resistance to EGFR-TKIs but also to characterize individual cell clusters and investigate the distinct resistance mechanisms exhibited by different cell populations [31]. Additionally, Kashima et al. [123] identified a subpopulation of small cells exhibiting elevated AURKA expression by analyzing transcriptomic changes in *EGFR*-mutant cells treated with gefitinib using scRNA-seq. AURKA encodes a kinase that enables *EGFR*-mutant cells to develop resistance to EGFR-TKIs [124].

Cellular heterogeneity is a key driver of drug resistance, as different subpopulations of cells may adopt distinct survival strategies. In cancer therapy, scRNA-seq proves invaluable in identifying the presence of distinct tumor subpopulations that may respond differently to various treatments, thereby serving as a foundation for detecting drug-resistant subpopulations [125]. For instance, Moghal et al. [126] revealed through scRNA-seq analysis of *EGFR*-driven lung adenocarcinoma xenograft tumors that DTPs might arise from pre-existing subpopulations of specific cancer cells. This analysis also demonstrated that EGFR-TKI treatment can activate certain pathways, ultimately leading to the emergence of drug resistance. Single-cell RNA sequencing studies of *EGFR*-mutant lung cancer have revealed that DTP cells display distinct gene expression profiles compared to the parental tumor cells. Notably, the expression of anti-apoptotic proteins, such as BCL2L1/BCL-XL, is significantly upregulated in DTP cells, enhancing their survival during drug treatment [38]. Similarly, Martinez-Marti et al. [127] applied single-cell transcriptome sequencing to evaluate the distribution of cellular clones in xenograft tumor samples from mice treated with afatinib or capmatinib, uncovering the pivotal role of *KRAS G12C* mutation-driven tumor subclones in targeted therapy resistance. Investigating these drug-resistant subclones could help mitigate the development of drug resistance and pave the way for novel strategies in tumor therapy. This advanced sequencing technique provides a high-resolution view of cellular heterogeneity, allowing researchers to identify diverse subpopulations of DTP cells, uncover their unique gene expression patterns closely tied to resistance mechanisms, and track transcriptional changes associated with the transition to a drug-tolerant state.

Single-cell RNA sequencing technology offers essential insights into identifying drug resistance mechanisms and devising therapeutic strategies by uncovering distinct tumor subpopulations and their varied responses to treatment. These advanced analyses enable a deeper understanding of tumor cell biology at the cellular level, highlight the pivotal role of tumor heterogeneity in drug resistance, and present potential cellular subpopulation profiles that can inform the development of personalized treatment regimens [128].

### 4.3. Identification of New Biomarkers of Drug Resistance

In investigating the mechanisms of drug resistance, researchers have employed scRNA-seq to identify and characterize various genes associated with resistance, revealing their impact on tumorigenesis, progression, and the efficacy of EGFR-TKI therapy.

Aissa et al. [129] analyzed cancer cell subpopulations in NSCLC tumor cells and tissues using scRNA-seq, uncovering mechanisms by which acquired drug resistance develops in NSCLC cell models and identifying biomarkers of resistance at the cellular subpopulation level. Their findings demonstrated that anti-apoptotic genes, along with the activation of the NF-κB and MAPK pathways, were present in early drug-resistant cells across different NSCLC cell lines, suggesting shared gene expression patterns during the initial stages of resistance. Consequently, selecting appropriate targeted drugs and employing them rationally may mitigate drug resistance in tumor subpopulations by downregulating specific survival mechanisms. Furthermore, in *EGFR*-mutant lung cancer cell lines, scRNA-seq analysis revealed changes in apoptosis-related gene expression following EGFR-TKI treatment. As resistance to EGFR-TKIs emerged, the expression of the *BCL2L1* gene was significantly elevated in drug-resistant cells, promoting survival. Notably, inhibiting BCL2L1 was shown to delay or overcome EGFR-TKI resistance [38]. In *EGFR*-mutant lung cancer, AXL, a receptor tyrosine kinase, plays a pivotal role in mechanisms of resistance to targeted therapy. Research indicates that during the development of resistance, the expression of AXL and its ligand, GAS6, is significantly upregulated, fostering the survival of resistant cells. AXL promotes the expression of low-fidelity DNA polymerases and enhances their activity through the activation of RAD18 [130]. These low-fidelity polymerases are more likely to introduce errors during DNA replication, establishing a genetic foundation for EGFR-TKI resistance. Moreover, single-cell RNA sequencing technology provides critical insights into the variability of AXL expression among tumor cells, stromal cells, and immune cells within the tumor microenvironment, aiding in the identification of potential resistance biomarkers. Kashima et al. [31] discovered that, in addition to known resistance genes such as *AURKA*, *VIM*, and *AXL*, the novel gene *CD74* played a critical role in EGFR-TKI resistance. Using scRNA-seq in clinical lung cancer tissue samples and EGFR-TKI-resistant cell models, they found that upregulation of CD74 inhibited apoptosis and induced acquired resistance to osimertinib, facilitating tumor regeneration. Hu et al. [131] demonstrated through single-cell transcriptional profiling of patient-derived xenografts from *EGFR*-mutated lung cancer patients that the neuroendocrine transcription factor *ASCL1* was upregulated following resistance to osimertinib treatment. ASCL1 likely conferred resistance by initiating an epithelial–mesenchymal gene expression program within a permissive cellular environment. Similarly, Lawal et al. [132], using NSCLC scRNA-seq datasets, reported that *MAP2K1*, *mTOR*, *YAP1*, and *EGFR* predominantly localized to monocytes, macrophages, Tregs, and CD8+ T cells, where they contributed to M2 polarization in the tumor microenvironment of both primary and metastatic NSCLC. These findings further suggest that these target genes play a pivotal role in tumor immune microenvironment remodeling. Additionally, Maynard et al. [48] identified significant upregulation of the WNT/β-catenin signaling pathway in lung epithelial cells of NSCLC patients with EGFR mutations. Their study suggested that targeting this signaling pathway could serve as a potential strategy to address resistance in EGFR-TKI treatment.

Overall, scRNA-seq technology has successfully identified several novel resistance biomarkers and potential therapeutic targets. These discoveries not only offer a more comprehensive understanding of drug resistance mechanisms but also lay a robust foundation for advancing personalized treatment strategies and developing innovative targeted therapies in the future.

In conclusion, scRNA-seq is widely applied in NSCLC. These applications provide valuable data to explore EGFR-TKI resistance mechanisms, develop new therapeutic strategies, and formulate personalized treatment plans. The key applications in this field are summarized in Table 1.

## 5. Potential Future Research Directions for scRNA-Seq in Lung Cancer Therapy

Single-cell RNA sequencing technology has revolutionized our knowledge of NSCLC, providing unprecedented insights, especially in studying the tumor microenvironment and tumor cell heterogeneity. By delving deeply into the complexities of lung cancer, researchers can identify cell subpopulations responsible for treatment resistance, uncover the diverse characteristics of tumors, discover novel biomarkers, and gain a more comprehensive understanding of the mechanisms driving resistance to therapy.

In lung cancer research, multi-omics integration strategies have demonstrated extraordinary effectiveness. For instance, one study [133] successfully identified key biomarkers associated with lung adenocarcinoma by combining genomic, transcriptomic, and proteomic data. Researchers conducted ultra-deep macrogenomic sequencing, host transcriptomic sequencing, and proteomic analysis on samples from patients with early-stage lung adenocarcinoma. This approach uncovered complex interactions between the local microbial community and the host. By integrating multi-omics data, the study offered a more comprehensive understanding of the mechanisms underlying lung adenocarcinoma and provided a solid scientific foundation for personalized treatment strategies. Similarly, Zhang and colleagues [134] constructed a multi-omics map that integrated single-cell RNA sequencing data with thousands of publicly available bulk RNA sequencing datasets. This analysis revealed the selective amplification of lung adenocarcinomas and squamous lung carcinomas in specific subpopulations. Further investigation of key transcription factors and stage-specific genes uncovered their functional roles, offering valuable insights into potential targets for early diagnosis and treatment. In another milestone, Bernard et al. [120] created the first complete cellular atlas of lung cancer, a landmark achievement in the pursuit of personalized therapies. This atlas includes 52 distinct stromal cell subpopulations and 12 cancer cell subpopulations, providing an unprecedentedly detailed view of lung cancer and its tumor microenvironment. However, one limitation of scRNA-seq technology lies in the loss of spatial information during the suspension preparation process—a critical aspect for studying tumor microenvironments. In recent years, advancements in spatial transcriptomics have addressed this gap, enabling the study of spatial heterogeneity and enhancing our understanding of tumor biology [135,136]. Under selective pressure from EGFR-TKIs, single-cell RNA sequencing and spatial transcriptomics become indispensable tools for analyzing tumor cell composition, deciphering their spatial organization, and uncovering the molecular mechanisms underlying cell–cell interactions that sustain tumor heterogeneity.

To address the challenge of integrating single-cell gene expression data with high-throughput drug screening, researchers have developed a computational method capable of predicting tumor cell sensitivity to various drugs. This method identifies subpopulations of tumor cells that exhibit differential drug responses within scRNA-seq data. Beyondcell, a tool designed for this purpose, distinguishes between drug-resistant and drug-sensitive subpopulations by uncovering the heterogeneity in cellular and drug responses. It further proposes cancer-specific therapies, offering a more precise analysis for identifying EGFR-TKI resistant cells and investigating the underlying mechanisms of heterogeneity and drug resistance within tumors [137]. Additionally, a migration learning model has been introduced to identify drug sensitivity biomarkers by predicting single-cell drug responses. This model provides valuable insights into tumor heterogeneity and facilitates cell-resolution therapies [138]. Another notable advancement is scRank, a drug-responsive cell type inference method that can predict drug-responsive cell types directly from unprocessed scRNA-seq data. This method reveals the therapeutic mechanisms of various drugs and aids in the development of more precise treatment regimens [139]. Its application in medulloblastoma has demonstrated its accuracy in identifying tumor subtypes that are sensitive or resistant to the Smo inhibitor vismodegib. These innovative computational tools are poised to harness the full clinical potential of scRNA-seq data. They offer critical support for analyzing gene expression changes linked to EGFR-TKI resistance and tumor microenvironment heterogeneity, paving the way for a deeper understanding of the mechanisms underlying the onset and progression of NSCLC.

With the ongoing advancements in single-cell histology and multi-omics-integrated analysis techniques, researchers are now better equipped to precisely identify the molecular characteristics of patients’ tumors. This progress lays a solid foundation for the creation of personalized treatment plans, which not only enhance therapeutic effectiveness and minimize unnecessary side effects but also drive the development of more targeted treatment strategies. Ultimately, these efforts contribute to improving both the survival rates and treatment outcomes for lung cancer patients.

## 6. Conclusions

The rapid advancement of single-cell sequencing technology has offered unprecedented insights into the mechanisms underlying EGFR-TKI-acquired resistance in NSCLC. This cutting-edge technology allows for an in-depth exploration of tumor cell complexity and heterogeneity, shedding light on the diverse gene expression patterns linked to resistance and enhancing our understanding of how the tumor microenvironment contributes to EGFR-TKI resistance. Moreover, scRNA-seq excels in providing detailed analyses of changes within the tumor microenvironment, identifying subpopulations of drug-resistant cells, and uncovering novel biomarkers associated with drug resistance. These breakthroughs pave the way for more comprehensive investigations into the mechanisms of acquired resistance to EGFR-TKIs. Additionally, the progress in personalized therapy hinges on a thorough understanding of tumor heterogeneity. Single-cell sequencing facilitates more precise tumor characterization, serving as a crucial foundation for informed treatment decisions.

Although scRNA-seq technology holds significant potential, challenges persist due to the diversity of research findings and varying perspectives. Disparities in sample collection, experimental design, and data analysis methods across research teams often result in differing interpretations of drug resistance mechanisms. Consequently, integrating and harmonizing these findings will be vital for propelling the field forward. Future studies should emphasize the adoption of standardized methodologies and foster multicenter collaborations to enhance the reproducibility and comparability of results. Moreover, the application of single-cell sequencing should extend beyond basic research, with its potential in clinical practice actively pursued.

Overall, scRNA-seq offers significant promise in uncovering the mechanisms behind acquired resistance to EGFR-TKIs. Only through ongoing research and interdisciplinary collaboration can we effectively overcome the challenges associated with resistance to targeted therapies in NSCLC, ultimately providing patients with more precise treatment strategies and an enhanced quality of life.

## Figures and Tables

**Table 1:** Application of scRNA-seq to reveal the mechanisms of acquired resistance to EGFR-TKIs in NSCLC.

Applications	Category	Research Findings	Clinical Significance	References
TME	TAMs	ASLC40A1+ TAMs reshape the TME. Increase in immunosuppressive subtype.	✓ Establishing an immunosuppressive TME by reshaping the TME.	[12,116,117]
	CAFs	Promote EMT and enhance tumor invasion.	✓ Providing insights into the mechanisms of EGFR-TKI resistance.	[118]
	Neutrophils	CXCR1+ neutrophils activated the TNF-α/NF-κB signaling pathway.	✓ The formation of an immunosuppressive TME promotes EGFR-TKI resistance.	[119]
	Immune checkpoints	PD-L2 impaired CD8+ T cell-mediated apoptosis.	✓ Providing potential targets for overcoming resistance.	[85]
Heterogeneity	Biopsy specimens from advanced NSCLC patients	Revealing extensive inter-individual and intra-tumor heterogeneity. Highlighted the clonal evolution heterogeneity of tumor cells.	✓ Underscoring the potential of tailoring therapies to specific cellular subpopulations associated with resistance mechanisms.	[48,120,121,122]
	Drug-resistant cell line models	Characterized individual cell clusters. Identification of tumor subpopulations with distinct characteristics.	✓ Providing insights into the mechanisms of EGFR-TKI resistance.	[31,123,124]
	Lung adenocarcinoma xenograft tumors	Discovered drug-tolerant persister subpopulations. Identification of tumor subpopulations with distinct characteristics.	✓ Paving the way for novel strategies in tumor therapy.	[125,126,127]
Resistance biomarkers	Target Genes	Apoptosis-related gene *BCL2L1*. Identification of known resistance genes such as *AURKA*, *VIM*, and *AXL*, as well as the novel gene *CD74*. The neuroendocrine transcription factor *ASCL1*. *MAP2K1*, *mTOR*, *YAP1*, and *EGFR*.	✓ Providing potential biomarkers for accurate prognostic. ✓ Advancing personalized treatment strategies and developing innovative targeted therapies.	[31,129,130,131,132]
	Signaling pathways	NF-κB and MAPK pathways. WNT/β-catenin signaling pathway.	✓ Providing ideas and frameworks for the development of new treatment strategies.	[48,129]

Abbreviations: TME, tumor microenvironment; TAMs, tumor-associated macrophages; CAFs, cancer-associated fibroblasts; EMT, epithelial–mesenchymal transition; NSCLC, non-small cell lung cancer; EGFR-TKI, epidermal growth factor receptor tyrosine kinase inhibitor.

## Data Availability

No new data were created or analyzed in this study. Data sharing is not applicable to this article.

## Abbreviations

APOBEC	Apolipoprotein B mRNA-editing catalytic polypeptide-like
CAFs	Cancer-associated fibroblasts
CTLs	Cytotoxic T lymphocytes
DTP	Drug-tolerant persister
ECM	Extracellular matrix
EGFR	Epidermal growth factor receptor
EGFR-TKI	Epidermal growth factor receptor tyrosine kinase inhibitor
EMP-1	Epithelial membrane protein-1
EMT	Epithelial–mesenchymal transition
HGF	Hepatocyte growth factor
IARC	International Agency for Research on Cancer
NSCLC	Non-small cell lung cancer
SCLC	Small-cell lung cancer
ScRNA-seq	Single-cell RNA sequencing
ST	Spatial transcriptomics
TAMs	Tumor-associated macrophages
TME	Tumor microenvironment
Tregs	Regulatory T cells
